# A Low Cost Implantation Model in the Rat That Allows a Spatial Assessment of Angiogenesis

**DOI:** 10.3389/fbioe.2018.00003

**Published:** 2018-02-05

**Authors:** Paul Slezak, Cyrill Slezak, Joachim Hartinger, Andreas Herbert Teuschl, Sylvia Nürnberger, Heinz Redl, Rainer Mittermayr

**Affiliations:** ^1^Ludwig Boltzmann Institute for Experimental and Clinical Traumatology, Vienna, Austria; ^2^Utah Valley University, Orem, UT, United States; ^3^Department of Biochemical Engineering, University of Applied Sciences Technikum Wien, Vienna, Austria; ^4^Department of Trauma Surgery, Medical University of Vienna, Vienna, Austria

**Keywords:** fibrin, VEGF, animal models, angiogenesis, computational analysis

## Abstract

There is continual demand for animal models that allow a quantitative assessment of angiogenic properties of biomaterials, therapies, and pharmaceuticals. In its simplest form, this is done by subcutaneous material implantation and subsequent vessel counting which usually omits spatial data. We have refined an implantation model and paired it with a computational analytic routine which outputs not only vessel count but also vessel density, distribution, and vessel penetration depth, that relies on a centric vessel as a reference point. We have successfully validated our model by characterizing the angiogenic potential of a fibrin matrix in conjunction with recombinant human vascular endothelial growth factor (rhVEGF165). The inferior epigastric vascular pedicles of rats were sheathed with silicone tubes, which were subsequently filled with 0.2 ml of fibrin and different doses of rhVEGF165, centrically embedding the vessels. Over 4 weeks, tissue samples were harvested and subsequently immunohistologically stained and computationally analyzed. The model was able to detect variations over the angiogenic potentials of growth factor spiked fibrin matrices. Adding 20 ng of rhVEGF165 resulted in a significant increase in vasculature while 200 ng of rhVEGF165 did not improve vascular growth. Vascularized tissue volume increased during the first week and vascular density increased during the second week. Total vessel count increased significantly and exhibited a peak after 2 weeks which was followed by a resorption of vasculature by week 4. In summary, a simple implantation model to study *in vivo* vascularization with only a minimal workload attached was enhanced to include morphologic data of the emerging vascular tree.

## Background

The ability to understand and manipulate angiogenic processes has become a key element of many therapeutic approaches. Angiogenesis is an essential factor in wound healing and the backbone of any successful tissue engineering attempt (Li et al., 2003; Michlits et al., 2007; Rophael et al., 2007; Lovett et al., 2009; Buehrer et al., 2015). To study the complex system of biomaterials and growth factor stimuli that govern the process of angiogenesis, various *in vivo* and *in vitro* models have been developed (Norrby, 2006; Staton et al., 2009; Poulaki, 2011). Many animal models (Kneser et al., 2006; Arkudas et al., 2007; Malinda, 2009) rely on the concept of implanting biomatrices, which can be loaded with various growth factors or cells and allow the ingrowth of vascular structures. The emerging vasculature may then be tracked *via* techniques of varying technical complexity. These include *in vivo* perfusion with casting agents and subsequent scanning electron microscopy (Polykandriotis et al., 2008) of the resulting corrosion casts, magnetic resonance angiography (Polykandriotis et al., 2008), photoacoustic imaging (Meiburger et al., 2016), or *in vivo* perfusion with radiopaque contrast agents and *ex vivo* computer tomography scans (Sider et al., 2010). While these methods yield full three-dimensional representations of the vascular trees they usually do not succeed in detecting capillaries as contrast agent perfusion in the smallest of vessels is limited. To include these, histologic staining and counting of vessel cross sections is still commonly performed (Tilkorn et al., 2012; Lilja et al., 2013). The goal of this study was to combine an implantation based model of angiogenesis (Cronin et al., 2004; Rophael et al., 2007; Tilkorn et al., 2012; Lilja et al., 2013) that is time and work efficient with a computational analysis of manually marked vessels. This provides insight into the morphology of newly developed vascular structures that goes beyond simple vessel counting and does not require sophisticated technical equipment. We aim to facilitate the assessment of angiogenic properties of biomaterials and growth factors in conjunction with a characterization of the resulting vascularization, based on a spatial analysis of basic histologic slices, that results in an array of morphological read out parameters that provide a comprehensive representation of newly formed vasculature.

## Materials and Method

### Study Design

A modified implantation model of biomaterial filled silicone tubes (Cronin et al., 2004; Tilkorn et al., 2012; Lilja et al., 2013) around the inferior epigastric bundle in Sprague Dawley rats weighing between 350 and 450 g was used. The study design consisted of two parts: first, four groups of *N* = 10 samples were set up to study the angiogenic effect of recombinant human vascular endothelial growth factor (rhVEGF165) (PeproTech, Rocky Hill, NJ, USA) inside a fibrin matrix after a time period of 4 weeks. Second, three groups of *N* = 6 samples were set up to study the dynamics of vascularization in a fibrin matrix over time (baseline, 1 week, 2 weeks).

The growth factor treatment groups consisted of a low dose group [0.2 ml fibrin matrix (ARTISS, Baxter AG, Vienna, Austria) loaded with 20 ng rhVEGF165] and a high dose group (0.2 ml fibrin matrix loaded with 200 ng rhVEGF165). A carrier group received 0.2 ml fibrin matrix without the addition of rhVEGF165. A control group received tubes with no biomaterial filling.

The additional groups evaluating the dynamics of the level of vascularization (*N* = 6 samples) received a fibrin matrix with no external growth factor added. Two of these groups consisted of animals sacrificed and evaluated after 1 or 2 weeks, respectively. The group representing the initial time point of surgery (baseline) consisted of animals, which were already dead at the time of implantation. The assessment of the final time point at week 4 was based on the data gained from the fibrin only group described earlier (*N* = 10 samples).

### Surgical Procedure

**Figure 1:**
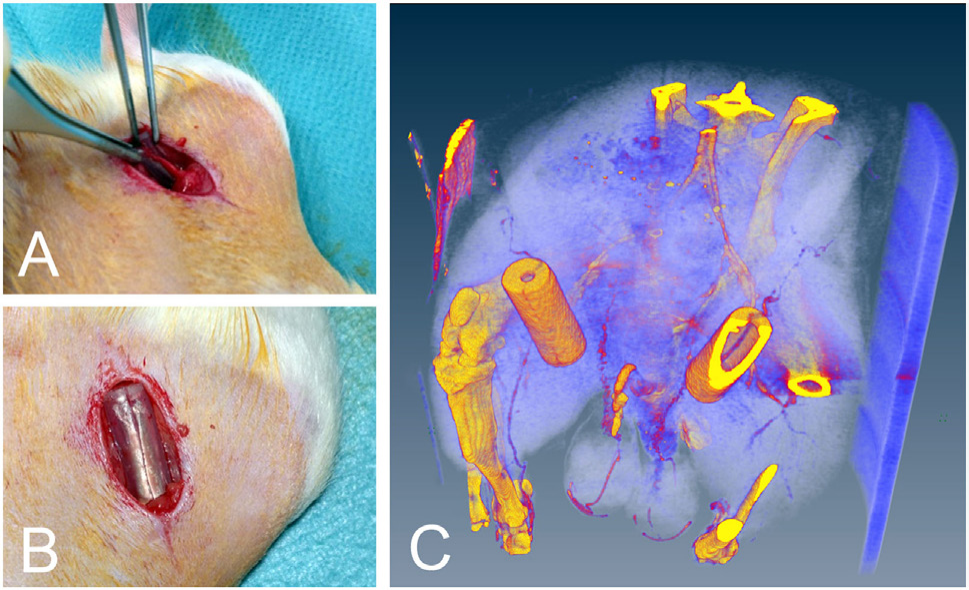
**(A)** Location of the vascular bundle before implantation. **(B)** Implanted silicone tubes. **(C)** Silicone tube *in situ* micro CT image. Note the perfused epigastric vascular bundle passing through the tube. The CT scan was performed in a single animal only to visually present the implantation and was not included in the analysis.

The animals were anesthetized with isoflurane 3% (Forane, Baxter AG, Vienna, Austria) while analgesia was provided by a single shot of buprenorphin, 0.01 mg/kg (Buprenovet, Bayer, Leverkusen, Germany) and meloxicam, 0.1 mg/kg (Metacam, Boeringer Ingelheim, Germany), daily, for a period of 3 days. The animal’s inguinal region was shaven and a longitudinal incision of approximately 1.5 cm was made bilaterally (Figure 1). The branching of the inferior epigastric bundle from the femoral bundle was exposed and the epigastric vessel trunk was carefully dissected and isolated on each side. Thereafter, custom made silicone tubes were placed around the vascular bundle in order to create a protected niche within the silicone shielding, centrically harboring artery, vein, and nerve. This provided a standardized volume for angiogenesis, in which newly formed vascular structures could be attributed to the specific local conditions and vascular ingrowth from surrounding tissue can be ruled out.

### Silicone Tubes

The implanted medical grade silicone tubes measured 1.5 cm in length, with an outer diameter of 6 mm and a wall thickness of 1 mm (Figure 1). A silicone membrane was glued onto each end of the tube, with a central aperture, allowing entry of the vascular bundle. The tubes were cut open on their sides in order to slip them around the vascular bundle. Once *in situ*, the tubes were closed with surgical sutures and filled with fibrin matrix and growth factors according to the group setup. They then remained inside the animals for up to 4 weeks before being harvested and analyzed. The implanted tubes were well tolerated by the animals, which showed no signs of pain, discomfort, or physical impairment. Further, no effect on gait was observed.

### Histologic Analysis

**Figure 2:**
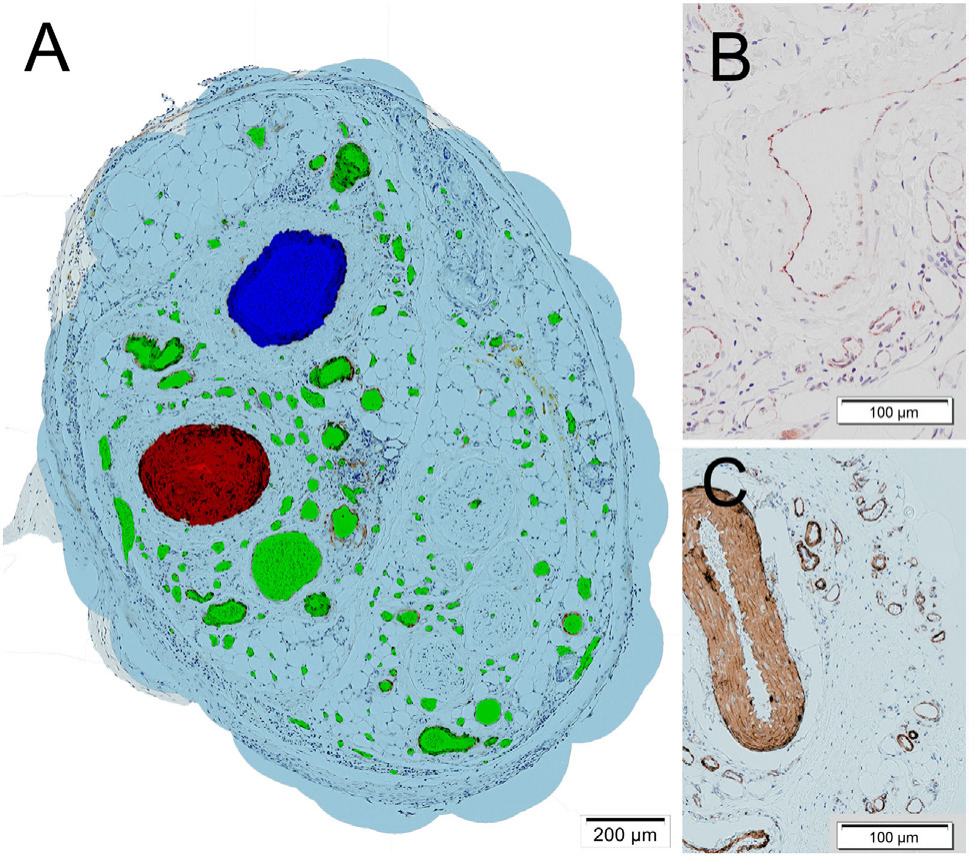
**(A)** Histologic section of a tissue sample obtained from one of the fibrin matrix filled tubes after 4 weeks. The cross sections of vessels were marked manually. The epigastric vein is marked in blue, the epigastric artery in red, other vessels including *de novo* capillaries in green. A virtual oxygen perfusion radius of 150 µm (Lovett et al., 2009) was marked in light blue around each vessel to illustrate and define an area which could potentially be supported by the vascular tree. **(B)** Detail of cluster of differentiation 31 stained vessels. **(C)** Detail of smooth muscle actin stained vessels.

Each animal yielded two cylindrical tissue samples which were extracted immediately after euthanization. They were fixed with 4% neutral buffered formalin and after 24 h rinsed in water for 1 h. The samples were then transferred into 50% ethanol for 1 h and stored in 70% ethanol. Dehydration was completed with further increase of the graded series of alcohol and embedded in paraffin *via* the intermedium xylol. The samples were sectioned in a plane perpendicular to the central axis at the middle of the harvested tissue cylinder at a thickness of 3–4 µm, resulting in circular slices with a centric artery and vein. They were subsequently deparaffinized and stained immunohistochemically for smooth muscle actin (SMA) and the cluster of differentiation 31 (CD31). SMA identifies the contractile cytoskeleton in the pericytes of mature vessels while CD31 stains endothelial cells and therefore additionally identifies emerging vessels and small capillaries (Helfrich et al., 2010) (Figure 2). Sections were prepared by blocking the endogenous peroxides with 3% hydrogen peroxide and antibody retrieval with steaming at pH 9.0 in HIER-T EDTA Puffer (ZUC029, Zytomed) or Dako Target Retrieval Solution pH 9.0 (S2367), respectively. SMA antibody (A 2547, Sigma) was then incubated in 1:5,000 and CD31 antibody (sc1506-R Santa Cruz) 1:100, both for 1 h. As secondary antibody, a polymer labeled antibody was used (ImmPRESS™, Vector Laboratories, Burlingame, CA, USA) and incubated for 30 min. Visualization was done with a peroxidase substrate kit (NovaRED™, Vector Laboratories, Burlingame, CA, USA) and counterstaining with hematoxylin.

An Olympus BX61VS scanning microscope was employed to obtain high resolution, full size scans of the samples at a resolution of 644 nm per pixel at a magnification of 200×.

### Image Analysis

On the obtained high resolution pictures, all vessel cross section were marked manually in their full extent by experienced researchers in a commercial image editor and color coded to enable automated vessel type detection (central artery red, central vein blue, capillaries green) (Figure 2). A vascular wall stained with either SMA or CD31 with a visible lumen or the presence of erythrocytes was used to identify vessel cross sections. The vessel markings were then automatically analyzed using the freely available software tool cell profiler (Carpenter et al., 2006), applying a detection and measuring pipeline in order to detect geometric shape, size, and location of the vessel cross sections.

The tricolored vessel markings were resampled to a size of 2,500 × 2,500 pixels and a color split conversion was performed to produce images only featuring the markings of a specific vessel type. The subsequent standard cell profiler object detection routine was then able to detect the marking of each vessel at 100% accuracy. Vessel center position, vessel area, and vessel major axis lengths were measured. In order to obtain a virtual estimate of perfusion, all detected vessels were then expanded by a radius of 150 µm (Lovett et al., 2009), which was executed within the same analytical routine. Finally, all obtained data were exported and further processed in a custom made Mathematica workflow for image identification, group allocation, and numerical analysis of the measurements including a statistical analysis.

### Multi Parameter Analysis

- Total vessel count—the total vessel count represents the total amount of vessel cross sections found in each sample, excluding the central artery and central vein.
- Total perfused area—to quantify the amount of tissue that could potentially be supported by the samples vascular network, a virtual perfusion area was introduced. The cross section of each detected vessels was expanded by a defined radius of 150 µm, representing the potential diffusion of oxygen into the tissue (Lovett et al., 2009). Overlapping perfused areas were single counted. The total of these areas was then calculated to obtain the final result. As such it is the area potentially perfused by the sum of all vessels found in the tissue cross section.
- Perfused area vessel density—the perfused area served as an approximate quantifier for the extent of the tissue surrounding the vascular tree, which in return permitted the calculation of vessels density within this tissue area. Each sample’s perfused area vessel density was calculated individually as the ratio of total vessel count and total perfused area.
- Vessel ingrowth distance—to assess the morphology of each vascular tree, the penetration depth of each vessel cross section into the scaffold was calculated. This parameter was obtained by measuring the linear distance from each marked vessels center to the central vein’s center. For this calculation, it was assumed that the vessels origin is the central vein according to Polykandriotis et al. (2008). This represents the depth of vascular penetration into the tissue.
- Vessel diameter—to further analyze vascular morphology, we determined the distribution of the vascular diameters in each sample. In our analysis, the vessels diameter was defined to be the minor axis of a vessel’s marked elliptical cross sections, including the vascular wall. This assured a representative diameter in oblique and elliptical cross sections of vessels and minimized the effects of compression artifacts.

#### Statistical Method

Throughout this paper, all statistical comparisons of means between experimental groups employed one-way analysis of variance (ANOVA) at a 95% confidence level (α = 0.05). *Post hoc* comparisons were conducted using a Duncan comparison test with figures indicating level of significance by the number of *’s (*p* = 0.05, 0.01, and 0.001). All uncertainty estimates are SEM.

## Results

### Vessel Growth Dynamics

#### Vessel Count and Perfused Area

**Figure 3:**
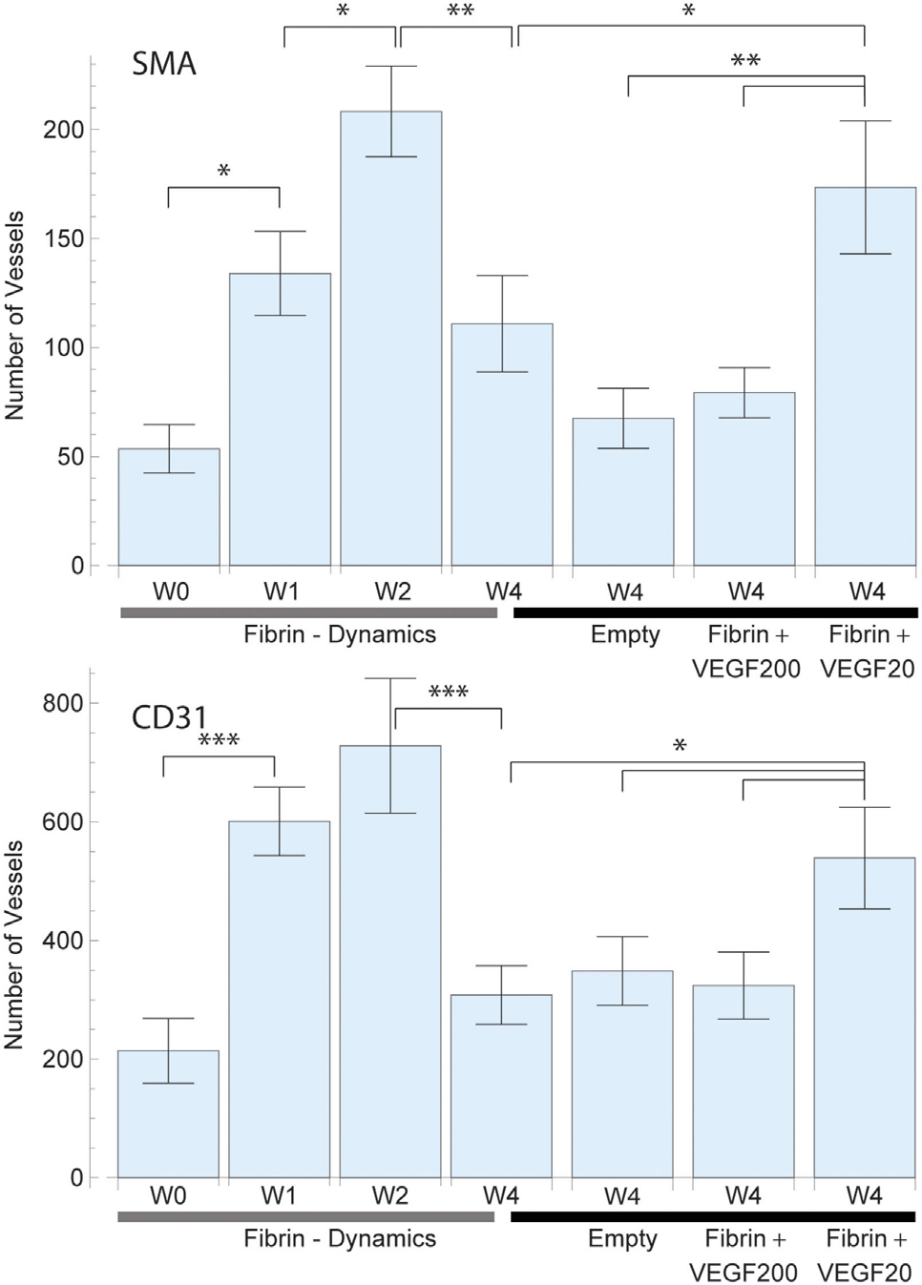
Average total vessel count per sample in each of the study groups for smooth muscle actin (SMA) (top) and CD31 (bottom) staining. The left 4 bars indicate the vascular growth in pure fibrin over a period of 4 weeks. A constant increase in vascularization was observed up to week 2, followed by vascular resorption. The right 4 bars indicate the effect of high dose (200 ng) and low dose (20 ng) recombinant human vascular endothelial growth factor (rhVEGF165) in a fibrin matrix in comparison to pure fibrin and no matrix. A statistical significant difference between the high and low dose rhVEGF165 group was detected. 20 ng rhVEGF165 yielded the best results, while 200 ng was comparable to the empty control.

**Figure 4:**
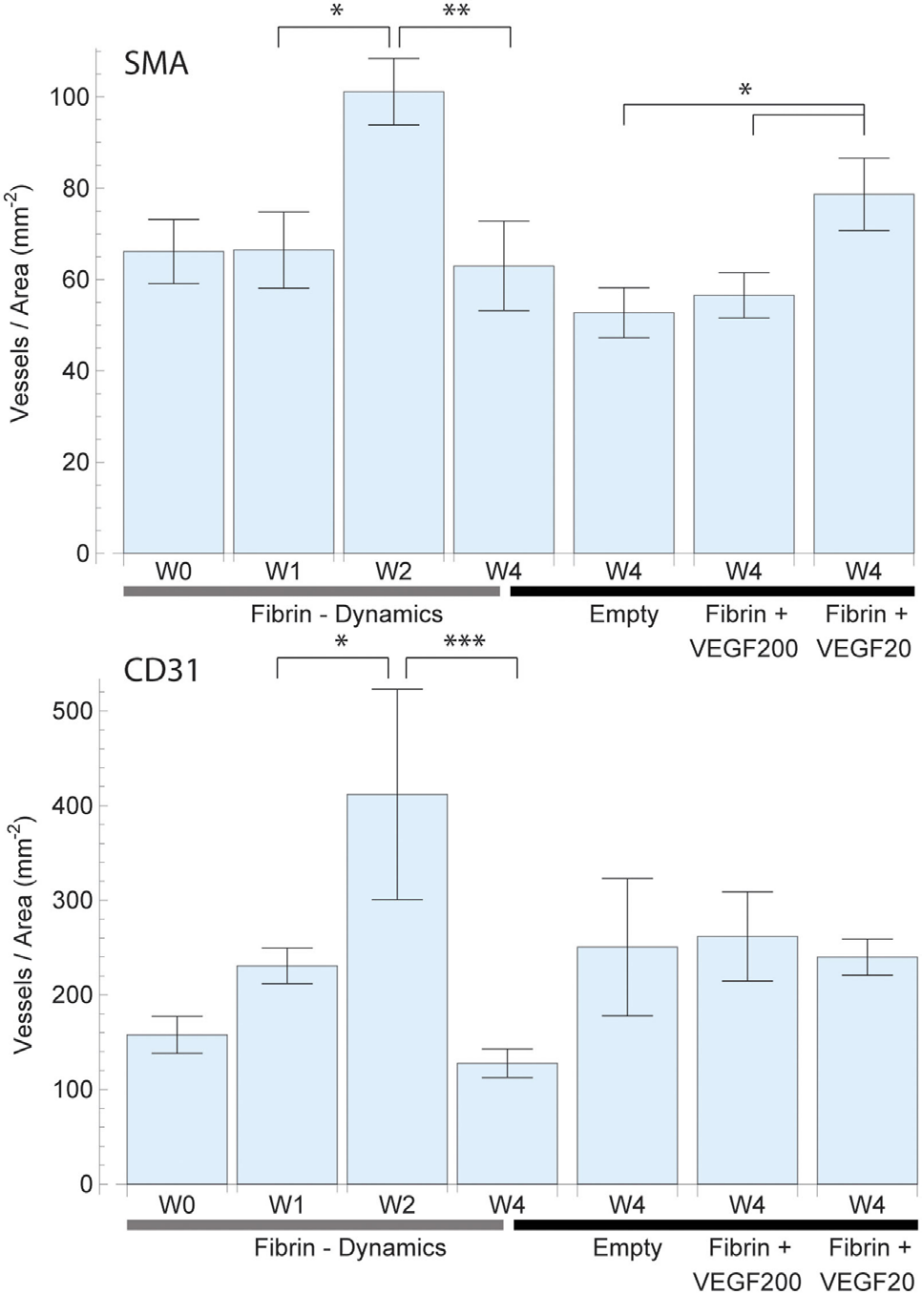
Mean vessel density based on the vascularized area of each individual sample for smooth muscle actin (SMA) (top) and CD31 (bottom) staining. The total vascularized area was calculated by expanding and adding up each vessel cross section by a defined radius of 150 µm representing the potential diffusion of oxygen into the tissue. Overlapping areas were single counted. The left 4 bars indicate the change in vascular density in fibrin over a period of 4 weeks, which peaked at week 2. The right 4 bars indicate the effect of high dose (200 ng) and low dose (20 ng) recombinant human vascular endothelial growth factor (rhVEGF165) in a fibrin matrix in comparison to pure fibrin and no matrix. The 20 ng rhVEGF165 group yielded the best results and a statistical significant difference to the empty control group was detected.

**Figure 5:**
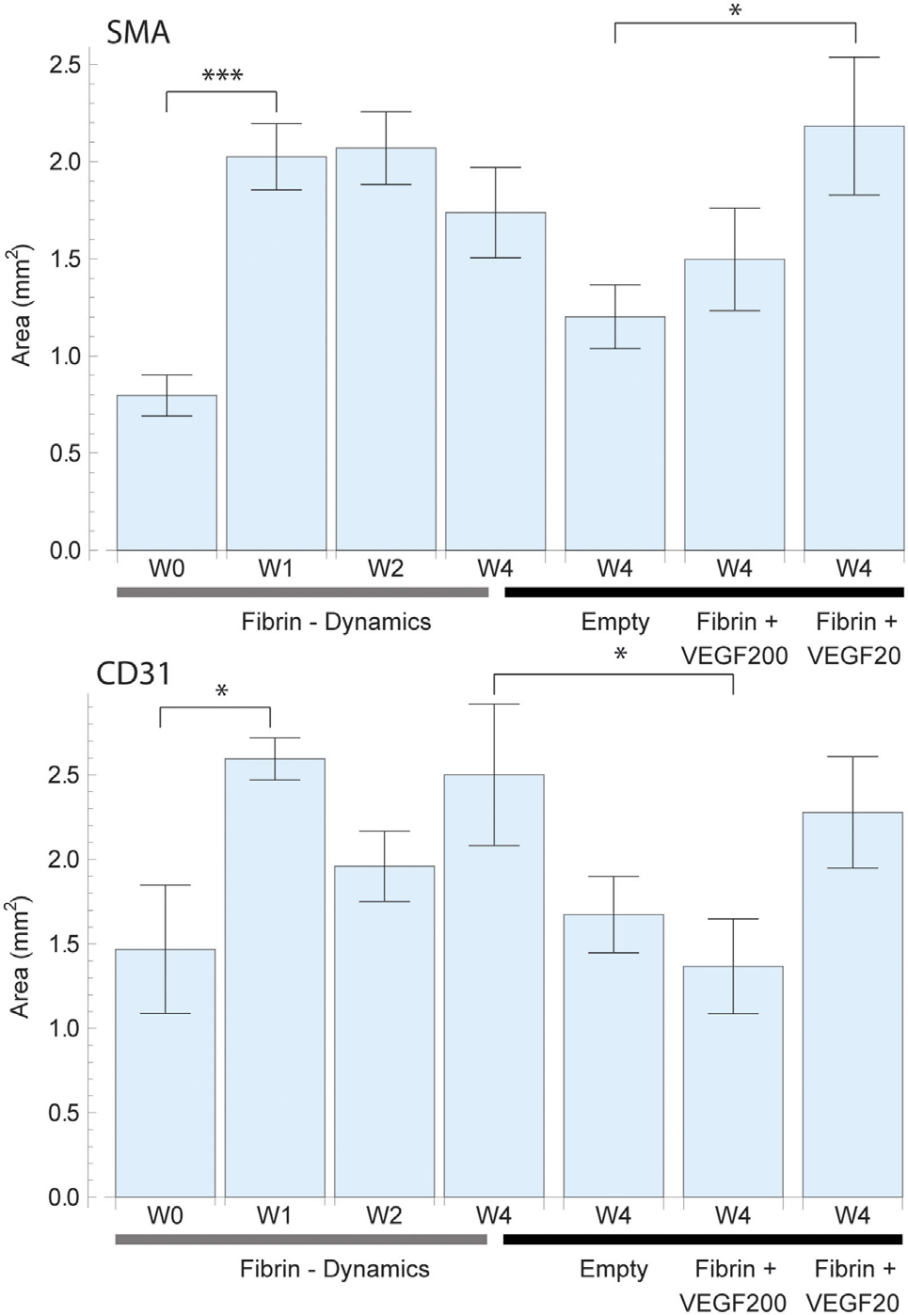
Total perfused area based on a virtual perfusion distance of oxygen which was defined as 150 µm around each vessel for smooth muscle actin (SMA) (top) and CD31 (bottom) staining. The perfused area was calculated by expanding adding up each vessel cross section by 150 µm. Overlapping areas were single counted. The left 4 bars indicate the change in perfused area in pure fibrin over a period of 4 weeks. There was an initially strong increase in vascularized area which then remained constant. The right 4 bars indicate the effect of high dose (200 ng) and low dose (20 ng) recombinant human vascular endothelial growth factor (rhVEGF165) in a fibrin matrix in comparison to pure fibrin and no matrix. The positive effects of fibrin and rhVEGF165 as well as missing effect of excessive rhVEGF165 dosings are confirmed once more.

The longitudinal collection of samples allowed for the consideration of the dynamical processes of angiogenesis (Figures 3–5). The implanted sample of week 0 showed a significant SMA (and CD31) stained increase [*F*(3,27) = 8.32, *p* = 0.001] {CD31: [*F*(3,27) = 11.37, *p* = 0.000]} in the number of 54 ± 11 (CD31: 214 ± 55) vessels over the first 2 weeks to a peak value of 208 ± 21 (CD31: 728 ± 114) (Figure 3). In the subsequent 2 weeks, we observed the effects of resorption reducing the vessel count to 111 ± 21 (CD31: 308 ± 49) at week 4. Further insight into the underlying dynamic processes was gained by considering the changes in vascularized vessel density and area (Figures 4 and 5).

The assessment of SMA vessel growth dynamics revealed a statistically significant [*F*(3,27) = 3.64, *p* = 027] increase in vasculature density to a peak of 101 ± 7 vessels/mm^2^ after 2 weeks which had remained constant over prior measurements at 66 ± 8 vessels/mm^2^ (Figure 4). We further observed that this increase in vascularization density was preceded by a significant [*F*(3,27) = 7.09, *p* = 0.001] increase in vascularized area during the first week from 0.80 ± 0.11 to 2.03 ± 0.17 mm^2^ (Figure 5), while no concurrent change was observed in vessel density. Subsequently, in the second week, the vascularized area remained constant, while the aforementioned vessel density peaked. Thereafter, we observed the onset of resorption of mature, SMA positive vasculature which is manifested in a significant decrease [*F*(3,27) = 3.64, *p* = 0.027] vessel density while largely maintaining the vascularized area in week 4 (63 ± 10 vessels/mm^2^ and 1.74 ± 0.23 mm^2^) (Figures 4 and 5).

Emerging, CD31-positive vascularization tells a similar story: an initial increase in vascularized area from 1.46 ± 0.40 to 2.60 ± 0.12 mm^2^ after 1 week that shows no significant change thereafter [*F*(3,27) = 1.97, *p* = 0.145] (Figure 5). Similarly, we observed no concurrent initial change in vascularization density after 1 week over the initial 158 ± 20 vessels/mm^2^. Subsequently however, we once again observed a significant spike [*F*(3,27) = 6.37, *p* = 0.002] to a peak density of 412 ± 111 vessels/mm^2^ at week 2 before the resorption drop in week 4 to 128 ± 15 vessels/mm^2^ (Figure 4).

#### Diameter and Ingrowth Distance

**Figure 6:**
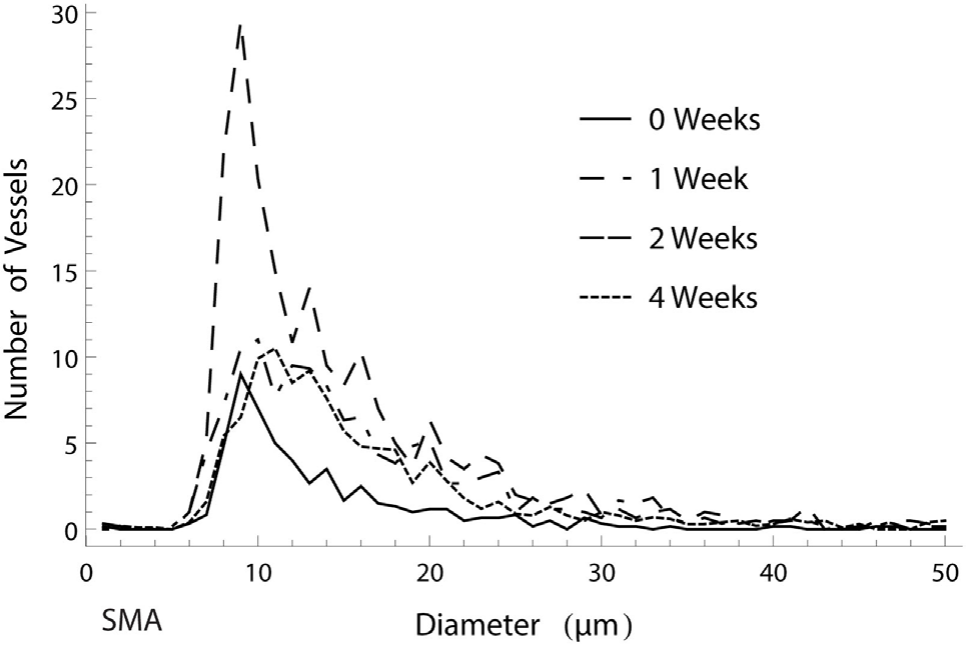
Histogram of the amount of smooth muscle actin (SMA) positive vessels of different diameters over the course of 4 weeks. The numerically largest gain in vessels was observed in week 2 in the capillary range.

The distribution of mature, SMA positive vessel diameters within the vascular tree remained unaltered over time. The numerically largest changes were observed in the capillary domain, as seen in Figure 6. When analyzing the ingrowth distances of the vessels, we saw a trend in accordance with the vascularized area results, with an initial increase in spread and a following decrease in penetration depth after 4 weeks as vascular resorption takes place.

### Growth Factor Effects

#### Total Vessel Count

Vessel detection was performed for both SMA and CD31 stained sections as shown in Figure 3. CD31 staining yielded a higher vessel count throughout the study as expected. After 4 weeks, the average vessel count in SMA stained sliced totaled 111 ± 22 (CD31: 308 ± 49) in the fibrin matrix group, while the empty control group totaled 68 ± 14 (CD31: 349 ± 58) (Figure 3). The addition of 20 ng of rhVEGF165 to the fibrin matrix resulted in an increase of the mean vessel count to 174 ± 31 (CD31: 539 ± 86). At the same time, adding 200 ng of rhVEGF165 diminished vascular growth and resulted in a vessel count of 79 ± 12 (CD31: 324 ± 56) (Figure 3). An ANOVA comparison indicates a statistically significant difference among the groups [*F*(3,39) = 5.17, *p* = 0.004] {CD31: [*F*(3,39) = 2.82, *p* = 0.05]}. A further comparison indicates the 20 ng rhVEGF165 group to be statistically different from the other tested groups.

#### Vessel Density and Total Perfused Area

The vessel density (Figure 4) and total perfused area (Figure 5) mirrored the trends observed in total vessel count in the SMA stained samples, indicating a similar beneficial effect of rhVEGF165. The addition of 20 ng rhVEGF165 resulted in a vessel density of 78 ± 8 vessels/mm^2^ (Figure 4) and a perfused area of 2.18 ± 0.35 mm^2^ (Figure 5). Again, high doses of rhVEGF165 appears to have no angiogenic impact as results in both vessel density of 56 ± 5 vessels/mm^2^ and perfused area of 1.50 ± 0.26 mm^2^ were in the range of the empty control group (density: 53 ± 5 vessels/mm^2^, area: 1.20 ± 0.16 mm^2^). No statistical significant difference in perfusion area and density were found [*F*(3,39) = 2.48, *p* = 0.07] and [*F*(3,39) = 2.45, *p* = 0.08].

Similar results were obtained for CD31: the addition of high doses of rhVEGF165 did not improve angiogenesis, resulting in a smaller perfusion area of 1.36 ± 0.28 mm^2^ over the fibrin matrix 2.50 ± 0.41 mm^2^ (Figure 5). No statistical significant difference in perfusion area and density were found [*F*(3,39) = 2.66, *p* = 0.06] and [*F*(3,39) = 1.91, *p* = 0.14].

#### Vessel Ingrowth

Concerning the morphology of the vascular tree, we were able to follow a trend that the use of a fibrin matrix, and even more so the addition of 20 ng rhVEGF165, would increase the ingrowth distance of mature, SMA positive vessels into the scaffold when compared to the empty control group (Figure 8). An equivalent behavior is observed in emerging, CD31 positive vessels.

#### Vessel Diameter

**Figure 7:**
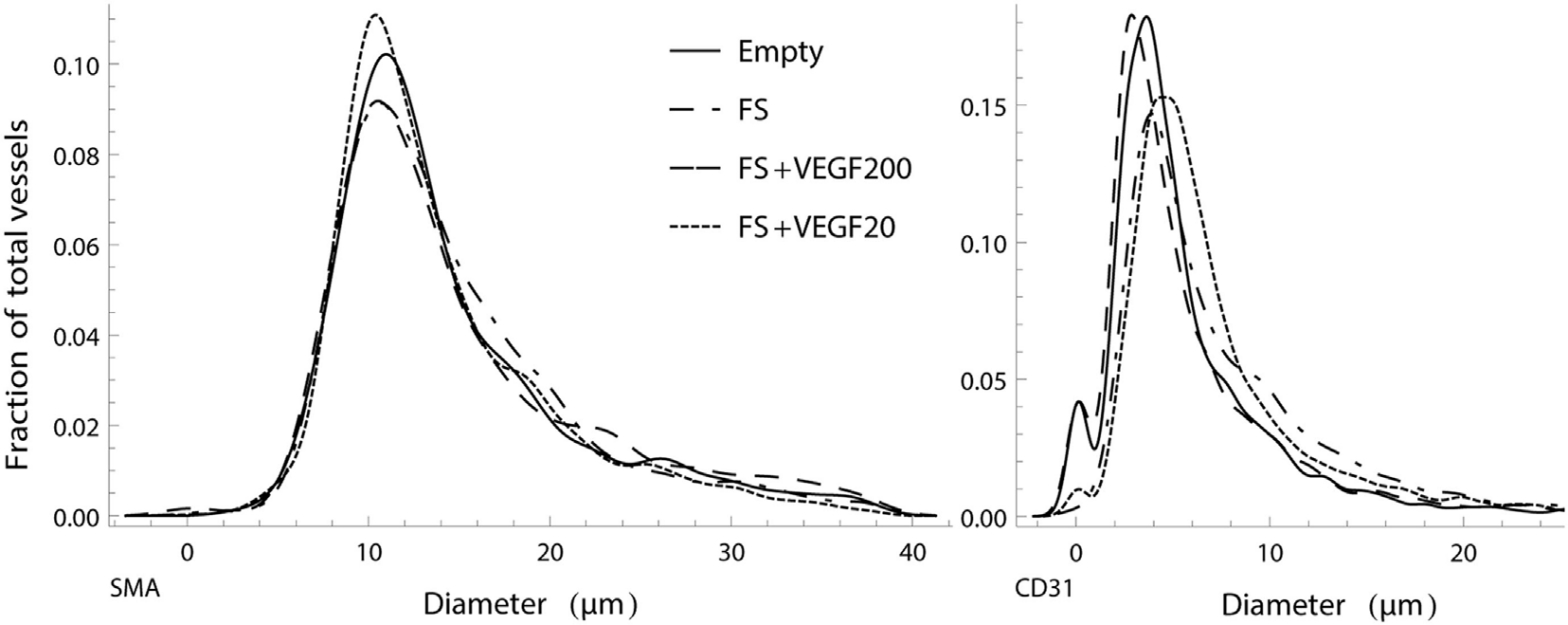
Normalized, smoothed histogram of vessel diameters as a fraction of the total vessels count for smooth muscle actin (SMA) (left) and CD31 (right) staining. The distribution of vessel diameters in the vascular tree is similar between the study groups and suggests that there is no strong influence of the different treatments in regard to ratio of larger to smaller vessels.

Analysis of both mature, SMA positive and emerging, CD31 positive vessel diameters showed that the different treatment options had no apparent influence on diameter distribution. While the numerically largest gain in vasculature was detected within the capillary diameter range, the ratio between larger and smaller vessels showed no strong alteration, with a proportionally increase in thicker vessels (Figure 7). Furthermore, the model clearly showed that CD31 stained vessels resulted in smaller average diameter than SMA stained vessel.

**Figure 8:**
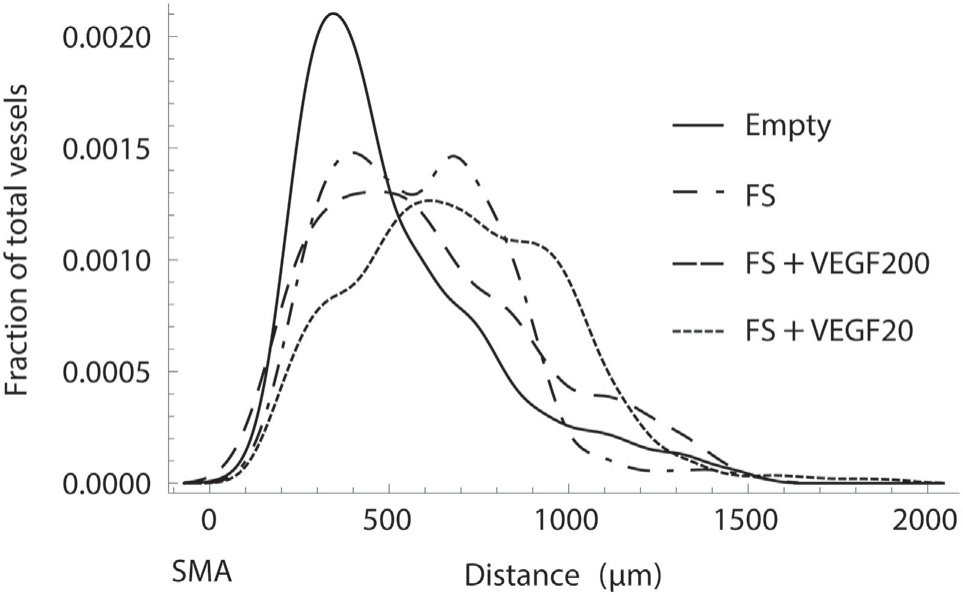
Normalized histogram of the transversal ingrowth distance of vessels into the scaffold as a fraction of the total vessels count. Fibrin with 20 ng of recombinant human vascular endothelial growth factor (rhVEGF165) resulted in the strongest ingrowth while it was severely limited in the absence of a fibrin matrix.

## Discussion

When establishing our model we meant to optimize it on two levels: first of all, we aimed for a comprehensive evaluation of vascular structures, going beyond a mere vessel count and second, we sought to keep it simple, efficient, and cost effective. Tanaka et al. (2003) have compared different vessel configurations inside implanted biomatrices and have outlined the benefits of an arteriovenous loop setup when creating larger tissue constructs. However, this would include microvascular anastomosis (Kneser et al., 2006; Arkudas et al., 2007; Buehrer et al., 2015) of small vessels and requires a considerable surgical effort. While this is worthwhile when aiming for large tissue quantities (Tanaka et al., 2003), in our analytical approach we favored the symmetrical tissue samples produced by a simple flow through configuration. This provided us with a standardized anatomic reference point (the central vein) needed to determine ingrowth distances. The measured main parameters (position, shape, diameter, vessel count) are then sufficient to calculate a satisfying diversity in parameters like vascular ingrowth distance, vessel density, and spatial distribution as well as diameter distribution, allowing potential comparisons between different growth factors, therapies, materials, and time points.

The model was refined to be a time efficient tool and seeks to keep all tasks involved as time economical and simple as possible. The surgical procedure requires no advanced micosurgical training or operative microscope and is swiftly performed within a few minutes per sample. Analytical procedures consist of basic immunohistologic staining and none of the steps involved, including vessel detection and microscopic scanning takes more than a few minutes per sample.

We validated our model, with a fibrin sealant as it is a frequently used and well-known matrix material (Wong et al., 2003; Arkudas et al., 2007; Mittermayr et al., 2008; Heher et al., 2017) and gives opportunity to portray its angiogenic effect as reference. We showed and detailed this angiogenic effect (Arkudas et al., 2007; Rophael et al., 2007; Mittermayr et al., 2008) in our model, which has led to an increase in newly formed vessels after 4 weeks compared to the control group. We further showed its positive effect in spatial parameters like vessel density, perfused area (which translates into vascularized tissue volume in 3D), vessel penetration depth and assessed vessel diameter. This information is problematic to obtain in standard subcutaneous implantations that lack a standardized reference point and where the specific origin of ingrowing vessels is unknown and where area and density calculations rely on predefined regions of interest which may introduce a certain bias. We also assessed the dynamics of vascularization based on these parameters, depicting a rather high initial vessel count immediately after surgery, which mostly consisted of vessels in the regime of capillaries that where in close proximity to the vascular bundle, which were not removable surgically. This was followed by a gradual outward growth of vessels and their subsequent resorption inside the fibrin biomatrix, in accordance with Arkudas et al. (2007) and Rophael et al. (2007). Through the spatial analysis, we were able to detail the specific parameters in which these changes took place: after 1 week, with the fibrin matrix still in place, we observed an increase in vasculature based on an increase in vascularized volume, while at the same time vessel density remained constant. At the 2 weeks time point however, with the fibrin matrix completely degraded, vascular growth was based on a constant vascularized volume and an increase in vessel density. We attribute this growth pattern to the scaffolding function of the fibrin matrix during the first week and to its ability to bind physiologic growth factors which affects vascular growth during the second week.

To further prove the model as a tool to assess advanced therapeutic effects, rhVEGF165 was added to the fibrin matrix and we validated its efficacy profile (Wong et al., 2003; Mittermayr et al., 2008). Adding 20 ng of rhVEGF165 resulted in an increase in vessel count as well as an increase in both, vascularized volume and vessel density when compared to pure fibrin, proving its angiogenic effect. Again, these changes were also represented in the spatial analysis of mature, SMA positive vessel distribution which revealed an increased ingrowth distance. The analysis of the vascular trees morphology further nicely showed that the numerically largest changes in vasculature took place within the capillary diameter range, with a distinct difference in total capillaries between the study groups. We also included a 10-fold higher dose of the growth factor into our study, in order to show physiological dose boundaries (Ozawa et al., 2004) and the ability of the model to detect such. Here, we saw a decrease in newly grown vasculature after 4 weeks when compared to the low dose rhVEGF165 group and the fibrin biomatrix group. Total vessel count dropped to levels comparable to the empty control group, as did vessel density and vascularized area. In general, SMA stained samples and CD31 stained samples yielded the same overall results, however, it is interesting to point out that for all similarities between SMA and CD31 results, specifically the resorption stage in particular is less likely to show differences in total perfused area in CD31 over SMA. This can be easily explained by the significantly larger number of detected CD31 vessels within a comparable area of potential oxygen diffusion which at this stage spans most of the sample. As such, any change in the number of CD31 vessels will not impact said area (due to the widely overlapping areas of the expanded vessels, data not shown) but will possibly result in a significantly different vessel density in SMA data.

In summary, we have refined an animal model of angiogenesis and tissue engineering to include a more detailed analysis and have validated it by characterizing the efficacy profile of a prominent angiogenic growth factor and a commonly used biomatrix. The setup and procedures involved proved to be efficient, economical and easy to execute, requiring only basic histologic techniques and the same workload attached as traditional vessel counting.

## Ethics Statement

Approval to this study by the Animal Protocol Review Board of the City of Vienna was obtained prior to conducting experiments.

## Author Contributions

PS conducted and designed the study, wrote the manuscript, conducted the surgeries, and performed data analysis. CS conducted data analysis and conceived and performed the analytical methods and wrote the manuscript. JH performed the surgeries and performed data analysis. AT provided material and participated in writing the manuscript as well as in the review process. SN established and conducted the histologic methods and provided scientific input during data analysis and interpretation. HR participated in the study design, data interpretation, the review process, and provided scientific input. RM conceived the study, participated in the surgeries, data interpretation, the review process, and provided scientific input.

## Conflict of Interest Statement

The authors declare that the research was conducted in the absence of any commercial or financial relationships that could be construed as a potential conflict of interest.
